# Profile of specific antibodies to the SARS-CoV-2

**DOI:** 10.1099/jmm.0.001335

**Published:** 2021-03-18

**Authors:** Danlei Mou, Huahua Feng, Ruiyuan Cao, Xing Weng, Lizhe Zhao, Lingling Yang, Ronghua Jin, Weijun Chen

**Affiliations:** ^1^​ Beijing Youan Hospital, Capital Medical University, Beijing, PR China; ^2^​ BGI PathoGenesis Pharmaceutical Technology, BGI-Shenzhen, Shenzhen, Guangdong, PR China; ^3^​ National Engineering Research Center for the Emergency Drug, Beijing Institute of Pharmacology and Toxicology, Beijing, PR China; ^4^​ BGI Education Center, University of Chinese Academy of Sciences, Beishan Industry Area 11, Shenzhen, PR China

**Keywords:** SARS-CoV-2, antibodies, Spike RBD protein

## Abstract

In this work, we studied the profile of IgM and IgG antibody responses to SARS-CoV-2 in 32 patients with COVID-19 from day 1 to day 24. IgM remained measurable for a much shorter period than IgG, suggesting that IgG antibody may represent the primary immune response.

## Introduction

A novel coronavirus called the severe acute respiratory syndrome coronavirus 2 (SARS-CoV-2), previously known by the provisional name 2019 novel coronavirus (2019-nCoV), has been identified as the causal agent of COVID-19 [1, 2]. SARS-CoV-2 is detectable in the blood, faeces and respiratory secretions of COVID-19 patients. Laboratory methods for SARS-CoV-2 diagnosis are primarily PCR-based to detect viral RNA in patient’s secretions/excretions and antibody-based to detect their immune responses, such as the litres of IgM and IgG antibodies in the blood. To understand the humoral immunity to this virus, we studied the profile of IgM and IgG antibody responses to SARS-CoV-2, as well viral RNAs, in throat swabs and anal swabs from 32 COVID-19 patients.

In our study, IgM and IgG antibodies were analysed by an indirect ELISA in 32 patients with COVID-19 from day 1 of their illness to day 24 every other 4 days and then every other 10 days. The general criteria for enrollment of patients were as follows: epidemiological history, body fever and Ct value of SARS-CoV-2 in the throat swab is less than 38. By the guidelines made by the Chinese National Health Commission, patients were classified into three categories: mild (without pneumonia), moderate or severe. A severe COVID-19 case was defined as a case with any one of the following criteria: (1) respiratory rate is at or above 30 per minute; (2) fingertip oxygen saturation is at or below 93 % at resting state; or (3) the ratio of the partial pressure of oxygen (PaO2) to the fraction of inspired oxygen (FiO2) (PaO2: FiO2) is at or below 300 mmHg. By the time of randomization, moderate patients were within the first 7 days from the onset of symptoms, and severe patients were within the 14 days from the onset of symptoms. The information of 32 patients is shown in Table 1. The median age of the mild patients was 42 years (interquartile range [IQR], 31 to 52 years); 65 % were female. The median age of the moderate patients was 62 years (interquartile range [IQR], 58 to 68 years); 100 % were female. The median age of the severe patients was 61 years (interquartile range [IQR], 58 to 68 years); 50 % were female. The 88 healthy contacts were also included in this study, whose information is shown in Table 2. The median age of the female healthy contacts was 34 years (interquartile range [IQR], 30 to 38 years). The median age of the male healthy contacts was 35 years (interquartile range [IQR], 30 to 40 years).

**Table 1.** Information of 32 patients

Characteristic	Mild	Moderate	Severe	Total
	(*N*=23)	(*N*=5)	(*N*=4)	(*N*=32)
**Age-yr**				
**Median (IQR)**	42 (31–52)	62 (58–68)	61 (58–68)	47 (33–58)
**Range**	25–59	52–70	48–69	25–70
**Female sex-no. (%)**	15 (65)	5 (100)	2 (50)	22 (69)

*The values shown are based on available data.

†Mild: symptomatic but not affecting daily activities; Moderate: symptomatic and slightly affects daily life; Severe: seriously affects daily life.

IQR, interquartile range; yr, year.

**Table 2.** Information of 88 healthy contacts

Characteristic	Female	Male	Total
	(*N*=53)	(*N*=35)	(*N*=88)
**Age-yr**			
**Median (IQR)**	34 (30–38)	35 (30–40)	34 (30–39)
**Range**	25–43	23–45	23–45

*The values shown are based on available data.

IQR, interquartile range; yr, year.

**Fig. 1.**
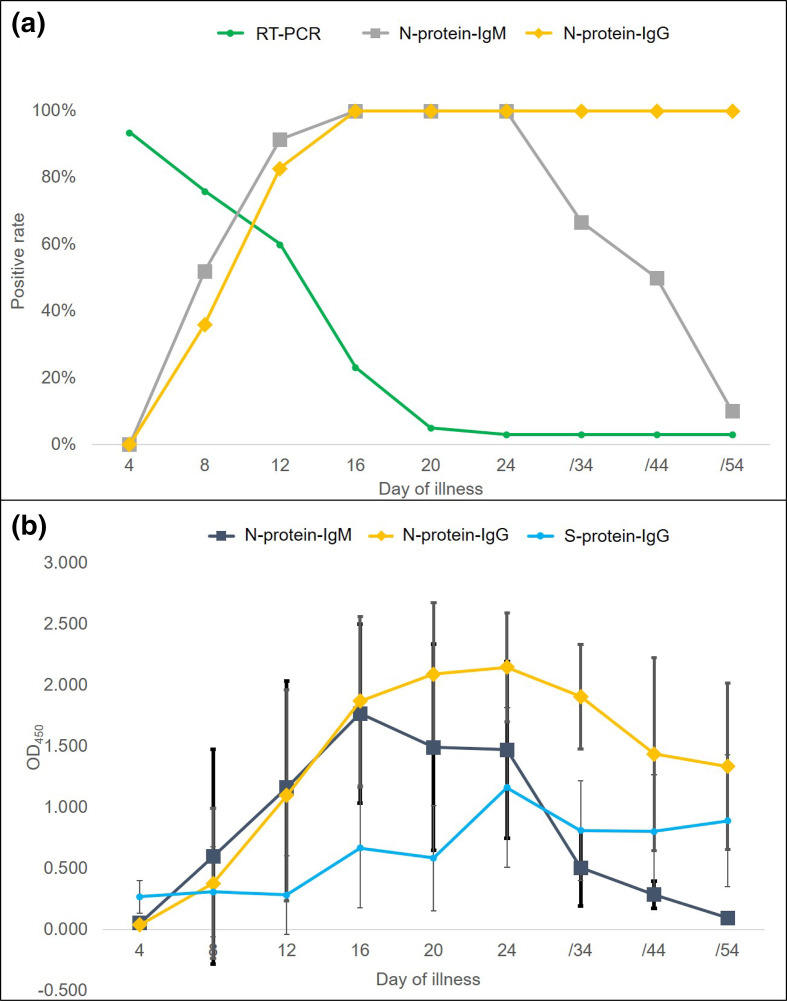
Profile of IgM and IgG antibody responses to SARS-CoV-2. (a) Positive rate of IgM and IgG antibody responses to SARS-CoV-2 and viral RNA of SARS-CoV-2 in the patients over the course of illness. (b) OD_450_ of IgM, IgG antibody responses to SARS-CoV-2 and IgG antibody to SARS-CoV-2 spike RBD protein in the patients over the course of illness.

SARS-CoV-2 IgM/IgG ELISA Kit (GBI, Beijing, PR China, catalogue no. 0601046) was used for detection and the antigen is the full length of N protein expressed from *Escherichia coli *. The specificity of the ELISA kit is 99 %, and the clinical sensitivity depends on the course of the disease. The IgG sensitivity for patients after 2 weeks of illness is around 100 %. All 32 patients tested negative for IgM and IgG at day 4 after the onset of symptoms. Of these patients, 18 tested positive for IgM and 12 tested positive for IgG at day 8, 29 tested positive for IgM and 26 tested positive for IgG at day 12. (Fig. 1a). All 32 patients were IgG-positive after day 16 and continued to have high levels of IgG up to 54 days after the onset of symptoms. The values of mean OD_450_ were calculated based on the results from positive patients. The IgG litres were low at the beginning of day 8 (mean OD_450_ value, 0.377±0.615, with the cutoff value 0.18 for a positive result), increased to an average of 1.099±0.863 at day 12, 1.871 at day 16, 2.090±0.585 at day 20, and peaked at 2.14±0.446 at day 24 and then maintain a high level. The IgM litres were low at the beginning of day 8 (mean OD_450_ value, 0.598±0.879, with the cutoff value 0.16 for a positive result), increased to an average of 1.162±0.874 at day 12, and peaked at 1.768±0.732 at day 16. This then declined to an average of 1.493±0.846 at day 20, 1.471±0.725 at day 24. All 88 healthy contacts tested negative for IgM and IgG. The specific antibodies to spike receptor binding domain (RBD) protein of SARS-CoV-2 were also tested using SARS-CoV-2 S1RBD IgG ELISA Kit (macro and micro-test, Jiangsu, PR China, catalogue no. 0601039), and the antigen was produced from insect cell. The specificity of the ELISA kit is 99 %, and the clinical sensitivity depends on the course of the disease. The sensitivity for patients after 2 weeks of illness is around 98 %. The IgG litres also peaked at 1.164±0.655 at day 24 and then declined (Fig. 1b).

Viral RNA was also tested by the throat swabs and anal swabs of these patients using real-time fluorescent RT-PCR kit for detecting SARS-CoV-2 (BGI Biotechnology, Wuhan, PR China, catalogue no. RM0349), which were proved with sensitivity of 100 copies/ml and specificity higher than 99.9 % with ORF1ab as the target. It revealed that 30 tested positive at day 4, 25 tested positive at day 8, 18 tested positive at day 12, 6 tested positive at day 16, 1 tested positive at day 20 and then this patient still tests positive in anal swabs but negative in throat swabs after day 24 to 54.

Our results suggest that throat viral RNA tested has more sensitivity during the acute or early phase, but it has 6.25 % false negative at least. However, 100% of patients had antibody responses to SARS-CoV-2 during the convalescent phase. The SARS-CoV-2 specific IgM remained measurable for a much shorter period, but the IgG antibody persisted for a long time, suggesting that IgG antibody to SARS-CoV-2 may represent the primary humoral immune response protecting patients against COVID-19. IgG began to decline from around the fourth week and failed to stabilize at a high level. The decline of RBD antibody may be related to the neutralizing antibody titre, which indicates that there is a potential for reinfection in patients with SARS-CoV-2 infection for a period of time after recovery. The profile of antibodies against SARS-CoV-2 was consistent with the findings of SARS-CoV infection [3]. The presence of high litres of IgG antibody to SARS-CoV-2 spike RBD protein in the patients at the convalescent stage also suggests that a S protein vaccine for active immunization and a concentrated human SARS-CoV-2 specific IgG antibody for passive immunization could be developed for the treatment of COVID-19. The profile of anti-SARS-CoV-2 antibodies may be helpful in the diagnosis and in epidemiologic surveys.
